# Phenotypic spectrum of dual diagnoses in developmental disorders

**DOI:** 10.1016/j.ajhg.2024.08.025

**Published:** 2024-09-30

**Authors:** Alys M. Ridsdale, Anna Dickerson, V. Kartik Chundru, Helen V. Firth, Caroline F. Wright

**Affiliations:** 1 Department of Clinical and Biomedical Sciences, Medical School, University of Exeter, St Luke’s Campus, Magdalen Road, Exeter EX1 2LU, UK; 2 East Anglian Medical Genetics Service, Clinical Genetics, Box 134, Addenbrooke’s Treatment Centre, Level 6, Addenbrooke’s Hospital, Hills Road, Cambridge CB2 0QQ, UK; 3 Wellcome Sanger Institute, Wellcome Trust Genome Campus, Hinxton, Saffron Walden CB10 1RQ, UK

## Abstract

As more patients receive genome-wide sequencing, the number of individuals diagnosed with multiple monogenic conditions is increasing. We sought to investigate the relative phenotypic contribution of dual diagnoses using both manual curation and computational approaches. First, we computed 1,003,236 semantic similarity scores for all possible pairs of 1,417 genes in the Developmental Disorder Gene2Phenotype (DDG2P) database using Human Phenotype Ontology terms. Next, for 62 probands with two molecular diagnoses in the Deciphering Developmental Disorders study, we computed semantic similarity scores between the probands’ phenotypes and DDG2P phenotypes associated with the two disorders and compared the results with manual attribution of proband phenotypes to none, one, or both of the genes. We found a spectrum of phenotypic similarity for dual diagnoses, both across all DDG2P genes and within dual diagnosed probands, from phenotypically distinct through blended to indistinguishable conditions. Pairwise semantic similarity scores between two DDG2P genes were a good predictor of the extent of phenotypic blending observed in probands. Dual diagnoses involving genes linked with synergistic phenotypes can result in more extreme presentations while those involving antagonistic phenotypes have spuriously high pairwise semantic similarity scores despite a potentially milder atypical presentation. We suggest that the phenotypic contribution of two molecular diagnoses may contain discrete, synergistic, or antagonistic elements. Conceptual recognition of this phenotypic spectrum is important for making a final clinico-molecular diagnosis and providing accurate genetic counseling.

## Teaser

Computational and manual assessment of dual diagnoses in developmental disorders reveals a spectrum of phenotypic similarity from distinct through blended to indistinguishable in which synergistic or antagonistic elements may result in absent or extreme phenotypes.

## Introduction

As exome and genome sequencing become widespread diagnostic tools, more and more patients and families with rare conditions are receiving genetic diagnoses. For example, ∼40% of children with severe developmental disorders can now be diagnosed using a genome-wide approach.^1^^,^^2^ This advance has resulted in challenges around variant interpretation and complexities resulting from dual diagnoses, where individuals have two separate monogenic conditions.^3^^,^^4^^,^^5^ Individuals with two (or more) monogenic conditions often pose a diagnostic conundrum for clinicians, as their phenotype may present as a novel mixture of two conditions with particular phenotypes arising from one, both, or neither causal variants. This confusion is exacerbated by the lack of conceptual framework or agreed descriptive terminology in the literature. A range of terms has been used to describe individuals with two molecular diagnoses, including dual, double, blended, distinct, overlapping, composite, obscured, and multilocus.^3^^,^^4^^,^^5^^,^^6^^,^^7^^,^^8^ These terms all describe a different phenomenon from digenic inheritance, where pathogenic variants in two interacting genes are required for a disease to manifest.^9^^,^^10^^,^^11^

Most published studies investigating dual diagnoses to date have been small, and a systematic theoretical evaluation of phenotypic overlap and comparison with large cohorts has not been performed. Here, we systematically investigate the similarity between pairs of different developmental disorders using the Deciphering Developmental Disorders (DDD) study and the Developmental Disorder Gene2Phenotype (DDG2P) database. G2P is a publicly accessible database designed for use in diagnostic variant filtering, which has been actively clinically curated since 2012.^12^ Each DDG2P entry associates an allelic requirement and a mutational consequence at a defined locus with a developmental disorder and confidence level.^13^ Using 62 DDD probands with two DDG2P diagnoses, we compare computational and manual approaches for attributing phenotypes to individual conditions and suggest a conceptual framework for describing the resulting phenotypic spectrum.

## Subjects and methods

### Calculation of theoretical pairwise semantic similarity scores

The DDG2P database was downloaded from https://www.ebi.ac.uk/gene2phenotype/ on October 10, 2022, and gene-disease associations with “strong” or “definitive” levels of evidence were retained.^13^ Human Phenotype Ontology (HPO) terms were cross-referenced against the HPO Consortium ontology index, and any obsolete HPO codes were updated or removed (https://raw.githubusercontent.com/obophenotype/human-phenotype-ontology/master/hp.obo).^14^ Where absent, manual curation of HPO terms was undertaken for genes with two diagnoses in the DDD study^1^ using HPO terms observed in ≥20% of overlapping affected individuals in DECIPHER. After this process, genes lacking any HPO codes were removed, and all remaining HPO codes for a gene were amalgamated for all instances of that gene in the database, and mode of inheritance terms were removed. Pairwise semantic similarity scores from 0 to 1 were calculated for gene pairs from HPO terms using the ontologySimilarity package (https://cran.r-project.org/web/packages/ontologySimilarity/vignettes/ontologySimilarity-introduction.html) in R Studio v4.2.1, which uses Lin’s expression of term similarity.^15^^,^^16^ Pairwise semantic similarity scores for genes with at least one opposing term were flagged based on a list of antagonistic HPO codes available from https://hpo.jax.org/app/; any pair of genes linked with both antagonistic terms was flagged as being antagonistic. Synergistic phenotypes were evaluated based on pairs of genes where both contained either ≥1 or ≥5 identical HPO terms.

### Selection and curation of DDD probands with dual diagnoses

The DDD study has UK Research Ethics Committee approval (10/H0305/83, granted by the Cambridge South REC, and GEN/284/12 granted by the Republic of Ireland REC). All probands underwent high-resolution exon-arrayCGH and exome sequencing; the full methods used in the DDD study have been described previously.^17^ A list of diagnosed probands was gathered from Wright et al.,^1^ using only “pathogenic” or “likely pathogenic” variants based on clinical assertion in DECIPHER (https://www.deciphergenomics.org/). Probands with a single diagnosis or >2 diagnoses were excluded, as were those with pathogenic multigenic structural variants and those with pathogenic variants in genes lacking HPO terms in DDG2P. Detailed manual curation of phenotypes in these probands was performed using DECIPHER. Phenotypes were attributed to conditions associated with none, one, or both contributing genes using OMIM,^18^ GeneReviews,^19^ and DECIPHER^20^ augmented with literature-based searches; phenotypes that were either common or subjective were excluded. The number of HPO terms attributed to neither gene was the number of HPO terms recorded for the proband minus any that were attributable to either one or both genes based on manual evaluation.

## Results

### Skewed bimodal distribution of pairwise semantic similarity scores for DDG2P genes suggests potential dual diagnoses could be distinct, blended, or indistinguishable

**Figure 1:**
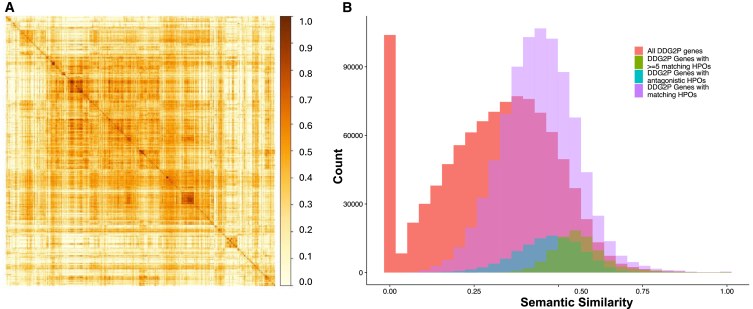
Pairwise semantic similarity scores between DDG2P genes (A) Heatmap of all pairwise semantic similarity scores for all 1,417 DDG2P genes. (B) Histogram of pairwise semantic similarity scores for all 1,417 DDG2P genes (red) and a subset with identical (≥1, purple and ≥5, green) or antagonistic (blue) HPO phenotypes. Semantic similarity scores were calculated using ontologySimilarity in R.

We downloaded the DDG2P database of 1,940 genes strongly linked with monogenic developmental disorders. Following targeted curation of genes containing diagnostic variants in the DDD study, we excluded any with zero HPO terms annotated in the database. Of the remaining 1,417 genes (Table S1), the mean number of HPO terms associated with each gene was 22 (range: 1–242), with higher numbers observed for pleiotropic genes associated with multiple different conditions. We then computed semantic similarity scores for all possible pairwise gene crosses (*n* = 1,003,236), using all HPO terms linked with each gene. The mean pairwise similarity score across all genes was 0.311 (Table 1), and no gene had a mean similarity score >0.5 across all of its pairwise crosses. Semantic similarity scores displayed a skewed bimodal distribution (Figure 1), suggesting that there are a sizable number of truly distinct conditions with no phenotypic overlap (i.e., scores near zero), as well as a larger number of conditions with increasingly similar phenotypes that may be clinically indistinguishable at the highest level of similarity. A comparable distribution was observed for smaller subsets of DDG2P genes containing either ≥1 diagnosis (∼690 genes) or ≥8 diagnoses (top ∼150 genes) in the DDD study, though with an increasing mean pairwise similarity score that may reflect ascertainment bias. We noted a weak positive association between the number of HPO terms associated with a gene and the average similarity score for that gene (log linear regression β = 0.11, *p* < 2e-16).

**Table 1:** Statistical summary of DDG2P pairwise semantic similarity scores

**Gene subsets**	**No. of genes**	**No. of pairs**	**Mean (SD) pairwise similarity score**	**Minimum similarity** **score**
DDG2P genes with HPO terms assigned	1,417	1,003,236	0.311 (0.174)	0.004
DDG2P genes with ≥1 diagnosis in the DDD study	690	237,705	0.370 (0.175)	0.004
DDG2P genes with ≥8 diagnoses in the DDD study	153	11,628	0.419 (0.166)	0.004
DDG2P genes with ≥1 identical phenotypes	1,417	851,776	0.443 (0.112)	0.032
DDG2P genes with ≥1 antagonistic phenotypes	905	114,548	0.471 (0.100)	0.062
DDG2P genes with ≥5 identical phenotypes	1,400	90,451	0.563 (0.087)	0.210

We also investigated the pairwise semantic similarity scores for all DDG2P genes associated with at least one identical or opposing (antagonistic) phenotypes. For both groups, we observed a much higher mean pairwise similarity score versus the entirety of DDG2P (0.44 and 0.47 for identical and antagonistic, respectively; Table 1). Notably, the scores were normally distributed around the mean (Figure 1) rather than having a bimodal distribution like the full DDG2P list, and there were no very low semantic similarity scores. This result is somewhat paradoxical for antagonistic phenotypes and suggests a potential issue in the way opposing phenotypes are handled by the scoring algorithm, i.e., the tree structure of HPO yields in a higher theoretical similarity score between phenotypes in closely related branches of the ontology regardless of whether they are opposing or not. For example, the terms “microcephaly” (HP: 0000252) and “macrocephaly” (HP: 0000256) have a high similarly score of 0.88 despite being opposite phenotypes, as they fall closely within the HPO tree under “abnormality of skull size.”

### Phenotypes in DDD probands with dual diagnoses can mostly be attributed to one or both genes, and higher pairwise semantic similarity scores correlate with increasingly blended phenotypes

We identified 121 probands (2.7% of those diagnosed) with composite diagnoses from the DDD study based on clinical assertion of variant pathogenicity.^1^ Of those where the inheritance was known, a third had one *de novo* variant and a third had two, as expected based on the high burden of *de novo* variants in developmental disorders. We excluded 59 from further analysis due to missing data (*n* = 23) or multigenic variants (*n* = 36), leaving 62 probands with two molecular diagnoses in DDG2P genes (Table 2). Probands in this cohort were 42% female, with a mean age of 7.8 years at recruitment and a total of 466 phenotypes (range: 1–21; median 7). We were able to manually attribute 428 phenotypes (92%) to either one (186; 43%) or both (242; 57%) genes in the dual diagnoses; 13 phenotypes were unattributable to either gene, and 25 were excluded on the grounds of being common or subjective.

**Table 2:** DDD probands with dual DDG2P diagnoses

**DECIPHER ID**	**Age (yrs) at recruitment**	**Sex**	**No. of HPO terms**	**Gene 1**	**Gene 2**	**Pairwise similarity score for gene 1 and gene 2**	**Similarity score for proband HPO terms versus gene 1**	**Similarity score for proband HPO terms versus gene 2**	**No. of proband HPO terms manually attributed to gene 1**	**No. of proband HPO terms manually attributed to gene 2**	**No. of proband HPO terms manually attributed to both genes**	**No. of proband HPO terms manually attributed to neither gene**	**No. of proband HPO terms (common/subjective) manually excluded**
258830	11.0	female	18	*TCF12*	*CDK13*	0.800	0.733	0.616	0	5	12	0	1
271137	11.0	male	9	*MBD5*	*CHD2*	0.743	0.686	0.672	1	0	7	0	1
291190	13.0	female	7	*SHANK2*	*PPP2R5D*	0.698	0.494	0.452	0	4	3	0	0
291341	10.0	female	6	*ZC4H2*	*SYNGAP1*	0.619	0.146	0.143	3	1	2	0	0
303270	19.0	female	6	*NF1*	*CBL*	0.619	0.621	0.611	0	0	6	0	0
270803	9.3	female	10	*BPTF*	*QRICH1*	0.615	0.525	0.451	2	0	8	0	0
261175	7.8	male	9	*FLNA*	*ZBTB20*	0.603	0.450	0.373	1	0	8	0	0
299681	4.5	male	2	*YWHAG*	*STAG1*	0.600	0.673	0.494	0	0	2	0	0
307458	2.1	male	4	*TAOK1*	*SLC6A1*	0.594	0.737	0.441	0	0	3	0	1
300981	2.3	male	10	*DNMT3A*	*PTEN*	0.593	0.553	0.502	1	0	6	0	3
286914	6.8	male	6	*POU3F3*	*EHMT1*	0.588	0.581	0.440	0	1	4	0	1
271406	7.2	male	2	*IQSEC2*	*SMC1A*	0.584	0.589	0.425	0	0	2	0	0
272920	3.8	male	3	*IQSEC2*	*SMC1A*	0.584	0.688	0.535	0	0	3	0	0
272921	8.4	male	1	*IQSEC2*	*SMC1A*	0.584	0.536	0.298	0	0	1	0	0
272922	9.9	male	2	*IQSEC2*	*SMC1A*	0.584	0.472	0.243	0	0	2	0	0
300851	0.8	male	6	*PPP2R5D*	*FGFR3*	0.583	0.554	0.495	1	0	5	0	0
295136	1.3	female	3	*EEF1A2*	*NF1*	0.579	0.404	0.331	0	1	1	1	0
266333	1.7	female	7	*SMARCA4*	*ANKRD11*	0.577	0.514	0.421	1	1	4	0	1
293170	3.1	male	2	*KIDINS220*	*CC2D2A*	0.572	0.610	0.593	0	1	1	0	0
276438	8.3	female	5	*NAA15*	*CHD3*	0.569	0.474	0.398	1	0	3	0	1
273503	11.0	male	6	*TAOK1*	*ZEB2*	0.551	0.731	0.445	2	2	2	0	0
278939	3.4	male	11	*PACS1*	*RAD21*	0.548	0.547	0.444	1	2	7	0	1
280956	12.0	female	11	*NFIX*	*SMARCA2*	0.545	0.476	0.386	1	2	7	1	0
271955	2.4	male	6	*SCN2A*	*TBL1XR1*	0.542	0.501	0.337	0	3	3	0	0
265387	5.6	male	7	*CTCF*	*FBN2*	0.529	0.493	0.212	4	0	3	0	0
300478	2.3	male	8	*PBX1*	*RAF1*	0.515	0.732	0.373	0	0	8	0	0
290989	4.1	male	21	*GNB2*	*NAA15*	0.497	0.763	0.538	2	4	11	0	4
264530	7.3	male	4	*ATRX*	*SETD5*	0.495	0.486	0.352	0	1	3	0	0
281373	22.0	female	7	*NAA15*	*PRMT7*	0.473	0.699	0.381	0	3	4	0	0
264597	10.0	female	14	*ANKRD11*	*PDHA1*	0.463	0.507	0.405	2	2	10	0	0
307561	7.5	male	6	*ATRX*	*MED13*	0.461	0.411	0.363	2	0	4	0	0
275085	16.0	male	21	*ADNP*	*EBF3*	0.454	0.464	0.356	5	0	16	0	0
306054	3.5	female	4	*COL1A1*	*IQSEC2*	0.446	0.541	0.407	2	0	2	0	0
269481	5.5	male	4	*OPHN1*	*HSPG2*	0.443	0.510	0.276	1	0	3	0	0
305998	6.4	male	11	*GRIN2A*	*SETD5*	0.441	0.547	0.538	1	7	3	0	0
272998	16.0	female	10	*SLC13A5*	*SETD5*	0.439	0.690	0.538	1	3	5	1	0
271765	2.9	male	7	*MED13L*	*DMD*	0.437	0.436	0.189	4	0	2	0	1
271952	8.3	female	6	*PTEN*	*SIN3A*	0.424	0.449	0.357	0	0	6	0	0
283972	20.0	female	12	*POLR1C*	*SAMHD1*	0.406	0.738	0.163	2	0	10	0	0
304477	16.0	female	11	*HDAC8*	*PAX8*	0.393	0.460	0.367	7	1	0	1	2
305580	6.4	female	8	*WDFY3*	*MAN1B1*	0.392	0.379	0.234	2	3	1	0	2
278908	9.5	male	5	*TRIP12*	*CDK13*	0.391	0.691	0.530	0	1	4	0	0
276430	2.6	male	4	*NRXN1*	*ASH1L*	0.379	0.461	0.218	0	1	3	0	0
280286	13.0	male	5	*KMT2E*	*TAB2*	0.358	0.395	0.370	2	0	2	1	0
259242	8.4	male	5	*PTCHD1*	*COL1A1*	0.334	0.768	0.340	2	1	1	0	1
264155	13.0	female	6	*NF1*	*ITPR1*	0.311	0.447	0.239	4	1	1	0	0
269952	6.8	female	7	*SRCAP*	*DCX*	0.305	0.309	0.307	3	1	3	0	0
260920	9.2	female	6	*SOX11*	*TRIP12*	0.294	0.504	0.353	1	0	5	0	0
264822	6.3	female	10	*MYCN*	*SETD1B*	0.294	0.364	0.347	1	3	6	0	0
286794	1.4	male	3	*SHH*	*STS*	0.290	0.610	0.061	2	0	1	0	0
265526	10.0	male	3	*ARMC9*	*BRIP1*	0.285	0.374	0.257	2	0	1	0	0
304171	1.8	male	6	*PTPN11*	*NRXN1*	0.256	0.391	0.202	3	0	3	0	0
266071	10.0	male	7	*TCF20*	*STS*	0.251	0.556	0.282	3	1	3	0	0
301569	4.7	male	10	*TBX4*	*DYNC1H1*	0.249	0.394	0.318	1	6	3	0	0
304140	5.3	male	4	*NGLY1*	*COL4A3*	0.249	0.619	0.172	1	1	1	0	1
284672	8.6	female	14	*NEB*	*LZTR1*	0.246	0.425	0.005	1	5	8	0	0
281387	1.3	male	11	*AIPL1*	*FGFR3*	0.223	0.514	0.505	4	7	0	0	0
269970	2.4	female	9	*SLC6A1*	*TNFRSF13B*	0.213	0.454	0.492	5	2	0	2	0
269973	2.4	female	8	*SLC6A1*	*TNFRSF13B*	0.213	0.526	0.413	5	1	0	2	0
276436	11.0	male	12	*SYN1*	*SLC4A1*	0.134	0.813	0.105	4	2	0	3	3
293597	10.0	female	6	*SPATA5*	*TSHR*	0.082	0.714	0.086	3	0	2	0	1
306313	13.0	female	12	*SETD5*	*COL4A3*	0.070	0.445	0.078	9	0	2	1	0

**Figure 2:**
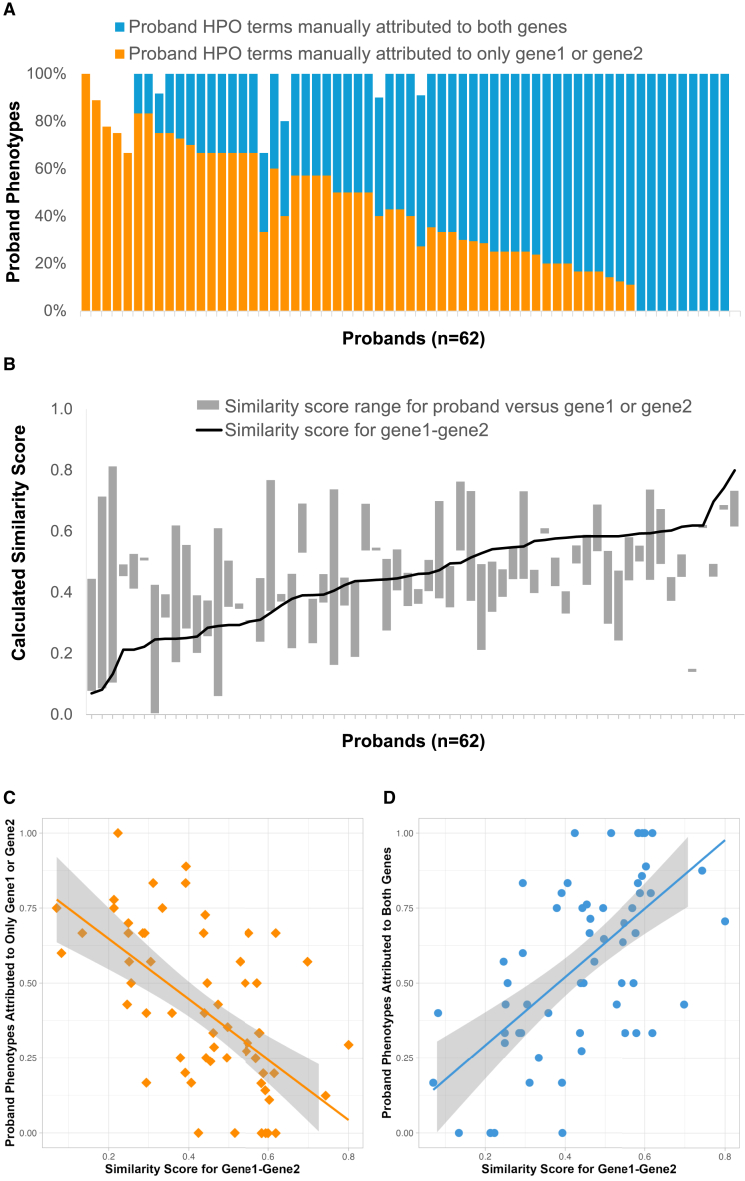
Comparison of manual and computational approaches gene-phenotype attribution in 62 DDD probands with dual diagnoses (A) Proportion of phenotypes (y axis) for individual probands (x axis) manually attributed to either one gene (orange), both genes (blue), or neither gene (white); ordered by increasing proportion of phenotype attributable to both genes. (B) Semantic similarity scores (y axis) for individual probands (x axis), comparing pairwise scores between the two genes (black line) with scores between each individual gene and the proband’s phenotype (top and bottom of gray boxes); ordered by increasing pairwise semantic similarity scores. (C) Proportion of a proband’s phenotype manually attributed to only one gene (y axis) versus pairwise semantic similarity scores for the two genes (x axis); linear regression performed in R, 95% confidence intervals shown. (D) Proportion of a proband’s phenotype manually attributed to both genes (y axis) versus pairwise semantic similarity scores for the two genes (x axis); linear regression performed in R, 95% confidence intervals shown.

Probands with dual diagnoses exhibited the full spectrum of phenotypic blending, ranging from distinct (five probands with phenotypes attributed solely to either gene) through various levels of blended to completely indistinguishable (nine probands with all phenotypes attributed to both genes) (Figure 2A). For example, one proband had two pathogenic *de novo* variants in genes linked with two distinct conditions, resulting in seven phenotypes attributable to *HDAC8* (Cornelia de Lange syndrome [MIM: 300882]) and just one attributable to *PAX8* (congenital hypothyroidism [MIM: 218700]). In contrast, another proband had two pathogenic variants in genes linked with highly overlapping conditions, and all their phenotypes were potentially attributable to either *NF1* (Neurofibromatosis-Noonan syndrome [MIM: 601321]) or *CBL* (Noonan syndrome-like disorder with or without juvenile myelomonocytic leukemia [MIM: 613563]). There was no significant correlation between the number of phenotypes attributed either to the genes or proband and the proportion attributed to either or both genes.

We next calculated semantic similarity scores between the probands' phenotypes and each of the individual genes in their dual diagnoses and compared this with the pairwise similarity score between those two genes (Figure 2B). There was a positive correlation between the probands' observed phenotypes and the individual semantic similarity scores for each individual gene in the dual diagnosis β = 0.35, *p* = 0.007). The difference between semantic similarity scores with the probands' phenotype and individual genes decreased as the pairwise similarity score between the two genes increased (linear regression, β = −0.44, *p* = 1.41e-05), suggesting that the presenting phenotype becomes more blended with increasing similarity. We found a surprisingly high concordance between manual and computational approaches. Pairwise semantic similarity scores between the two diagnostic genes were negatively correlated with the proportion of the probands’ phenotypes manually attributed solely to one gene (Figure 2C; β = -1.01, *p* = 2.98e-07) and positively correlated with the proportion manually attributed to both genes (Figure 2D; β = 1.14, *p* = 1.11e-07).

### Dual diagnoses involving genes linked with antagonistic phenotypes may have milder atypical presentations while those linked with synergistic phenotypes may be more severely affected

There was no overall difference in the number of HPO terms between individuals in the DDD study with dual versus single diagnoses (mean 7.4, *p* > 0.8). However, the five probands with all attributable phenotypes linked solely to one gene had more phenotypes than the others with dual diagnoses (mean 10.2, *p* < 0.05), suggesting a more diverse phenotype, while the nine with all attributable phenotypes linked to both genes had fewer (mean 3.8, *p* < 0.001). We hypothesized that some dual diagnoses might be less severe or have fewer phenotypes than single diagnoses due to the two conditions having antagonistic effects that cancel each other out, e.g., short versus tall stature. Mapping opposing phenotypes onto DDG2P gene pairs, we identified two DDD probands with partially antagonistic dual diagnoses in our cohort, both of whom fitted with our hypothesis: one had pathogenic variants in *ANKRD11* (linked with hypertelorism [MIM: 148050]) and *SMARCA4* (linked with hypotelorism [MIM: 614609]) but no evidence of abnormal eye morphology; the other had pathogenic variants in *HSPG2* (linked with micrognathia [MIM: 255800]) and *OPHN1* (linked with mandibular prognathia [MIM: 300486]) but no evidence of abnormal jaw morphology.

We also investigated dual diagnoses comprising genes linked with synergistic phenotypes where we hypothesized that the probands might have more pronounced phenotypes. Most conditions were linked with intellectual disability or global developmental delay for which severity is often not annotated. However, we identified several probands with dual diagnoses linked with quantitative synergistic phenotypes that were more pronounced than DDD probands diagnosed with either single condition. For example, one dual diagnosed proband had an occipital frontal circumference (OFC) of −6.83 standard deviations (SDs) and pathogenic variants in *ANKRD11* (MIM: 148050) and *PDHA1* (MIM: 312170), both of which are linked with microcephaly (mean OFC of −1.2 SD and −4.0 SD for DDD probands with single diagnoses in these two genes, respectively). Another proband had an OFC of +6.31 SD and pathogenic variants in *PPP2R5D* (MIM: 616355) and *FGFR3* (MIM: 602849)*,* both of which are linked with macrocephaly (mean OFC of +2.3 SD and +0.2 SD for DDD probands with single diagnoses in these two genes, respectively).

## Discussion

**Figure 3:**
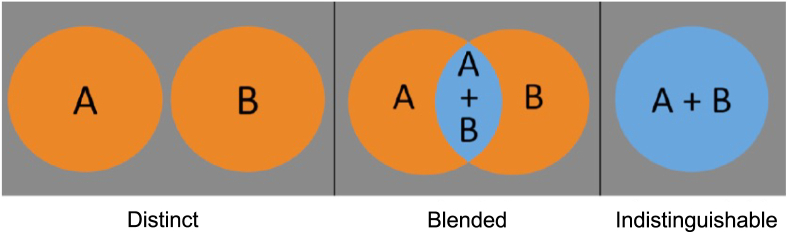
Conceptual overview of dual diagnoses Three classes of dual diagnoses are suggested, based on phenotypic overlap between two theoretical developmental disorders (A and B): distinct conditions (left) with no or very limited phenotypic overlap in which individual proband phenotypes may be clinically apportioned to one or other diagnosis; blended conditions (middle) with a moderate level of phenotypic overlap; and indistinguishable (right) with highly similar conditions in which individual proband phenotypes cannot be clinically apportioned into either diagnosis.

We have shown that dual genetic diagnoses make a small but important contribution to phenotypic diversity within monogenic developmental disorders. An individual proband with two molecular diagnoses may present with features that range from distinct phenotypes uniquely attributable to one or other gene, to overlapping phenotypes attributable to either gene that are ultimately indistinguishable when the two conditions become sufficiently similar (Figure 3). This complete spectrum was predicted using pairwise semantic similarity scores between DDG2P genes and recapitulated in probands with dual diagnoses in the DDD study and has not been fully articulated previously. We have also shown that dual diagnoses involving conditions linked with antagonistic phenotypes may result in absent phenotypes in a proband while those with synergistic phenotypes may result in more extreme phenotypes, either of which could make clinical confirmation of the diagnosis more challenging. Finally, we have shown that there is a linear correlation between the extent of phenotypic blending in the proband and the pairwise semantic similarity score between two genes contributing to a dual diagnosis.

Previous large-scale studies have found higher rates of dual (or more) diagnoses than we examined here. The most comprehensive to date identified 153 (8.5%) in 1,792 diagnosed probands with multiple potentially relevant genetic findings^5^; another identified 101 (4.9%) in 2,076 diagnosed probands and also showed that semantic similarity scores were significantly lower among probands in whom the phenotype resulted from two distinct disorders.^4^ We took a conservative approach to defining affected individuals, and included only 121 (2.7%) of 4,484 probands diagnosed with two genetic conditions by clinical assertion, which is likely to be an underestimate based on burden analyses.^21^ A further 561 (12.5%) of those with single diagnoses were thought to have only a partial diagnosis for their condition, suggesting potentially a second missing diagnosis. In addition, a total of 360 (6.5%) of 5,502 probands diagnosed based on automated variant classification in addition to clinical assertion were predicted to have a dual diagnosis.^1^ The true fraction of probands with dual monogenic diagnoses is unclear, and finding them depends heavily upon both the diagnostic workflow and the clinical assessment process. Some probands could justifiably be tested using multiple non-overlapping gene panels and are thus more likely to be diagnosed with several distinct conditions. Moreover, although the difference between dual diagnoses that constitute two independent conditions (in which each genotype alone is sufficient to cause disease) and those that are actually digenic (in which strictly both genotypes are dependent upon the other to cause disease) remains a relevant distinction, many apparently fully penetrant conditions may actually be incompletely penetrant and require additional variants for the condition to manifest. For some probands, it is plausible that two or more large-effect variants may be required to push the individual above a threshold for clinical presentation, consistent with an oligogenic disease model.^22^ In contrast, other probands may reach that same threshold with just one of those variants, depending upon other polygenic or environmental risk factors, consistent with a monogenic disease model with two independent diagnoses.

One unexpected finding of our study was the high calculated semantic similarity scores between opposing phenotypes. This effect is likely to be driven by the proximity of antagonistic phenotypes within the directed acyclic graph underlying the HPO, whereby two antagonistic terms may have the same parent term. However, this misleadingly high similarity presents a major limitation when clustering genes or probands based on HPO terms, as any true signal arising from phenotypic similarity may be obscured by apparent similarity between opposing phenotypes. Indeed, such similarity scores could drive spurious associations and point toward shared biology where none exists. In constrast, absent phenotypes resulting from antagonistic dual diagnoses likely results in lower similarity scores between the proband’s phenotypes and those linked with each individual condition. We therefore urge developers of semantic similarity scores to flag antagonistic phenotypes to alert users and implement an appropriate method to negate the proximity of the terms within the HPO tree and reduce the scores.

Our study has some important limitations. Most notably, we were forced to group phenotypes by gene to enable pairwise semantic similarity scores to be computed and compared against phenotypes in the DDD probands. While this had no effect for the majority of DDG2P genes that are only associated with one condition, it resulted in some pleiotropic genes being mis-represented in the dataset. Conditions caused by variants in the same gene with different modes of inheritance or pathomechanisms were grouped together despite sometimes resulting in completely different phenotypes. Although this issue could potentially be remedied by computing pairwise semantic similarity scores for gene-condition dyads,^23^ this approach would pose difficulties for the definition of discrete conditions in both the literature and DDD probands. “Lumping versus splitting” is an ongoing debate in genomic medicine,^24^ and both approaches have advantages and disadvantages. Focusing on genes also limited our ability to include multigenic structural variants associated with known syndromes, which are not currently included within DDG2P but meant that ∼30% of clinically asserted dual diagnoses in the DDD study were excluded. We were also limited by incomplete and inconsistent phenotyping, both of the DDD probands and the condition within the DDG2P database. We anticipate that the latter will be improved in future through the use of more systematic and automated curation of the literature.^25^

In conclusion, we have shown that phenotypes linked with pairs of developmental disorders lie on a spectrum of similarity from distinct through blended to indistinguishable. Individuals with dual molecular diagnoses may therefore present with broader or more pronounced phenotypes than those with either of the contributing single monogenic diagnoses. Semantic similarity scores between contributing pairs of genes may help determine the level of phenotypic blending. Importantly, probands with dual diagnoses linked with antagonistic phenotypes may have less severe or completely absent phenotypes, and their semantic similarity scores may be misleading. Our findings suggest that an objective similarity scale could be helpful for confirming diagnoses, determining the level of phenotypic overlap between different conditions and counseling patients about recurrence.

## Data and code availability

This study did not generate datasets.

## Acknowledgments

The authors are very grateful to the families, clinical teams, and scientists involved in the DDD study. The DDD study presents independent research commissioned by the Health Innovation Challenge Fund (HICF-1009-003), a parallel funding partnership between the 10.13039/100010269Wellcome Trust and the Department of Health, and the Wellcome Sanger Institute (WT098051). This research uses DECIPHER and was funded in part by the 10.13039/100004440Wellcome (226083/Z/22/Z). The study was supported by the National Institute for Health and Care Research Exeter Biomedical Research Centre and through the Comprehensive Clinical Research Network. The views expressed are those of the author(s) and not necessarily those of the Wellcome, NIHR, or the Department of Health and Social Care. For the purpose of open access, the author has applied a CC-BY public copyright license to any author accepted manuscript version arising from this submission.

## Declaration of interests

The authors declare no competing interests.

## Supplemental information

Table S1. Semantic similarity scores and HPO codes for included DDG2P genes
